# Endoscopic septum division to resolve nonstenotic gastric outlet obstruction after endoscopic submucosal dissection of the gastric antrum

**DOI:** 10.1055/a-2505-9309

**Published:** 2025-01-23

**Authors:** Chaoqin Wang, Suhuan Liao, Silin Huang, Ting Li, Yu Zhang, Jingzai Wang, RenJie Chang

**Affiliations:** 1 Department of Spleen and Stomach Diseases, Yunnan Provincial Hospital of Traditional Chinese Medicine, Medical School, Yunnan University of Chinese Medicine, YunNan, China; 2 701237 Department of Gastroenterology, South China Hospital, Medical School, Shenzhen University, Shenzhen, China; 3 Department of Gastroenterology, The First Peopleʼs Hospital of Yunnan Province, The Affliated Hospital of Kunming University of Science and Technology, YunNan, China

A 68-year-old female patient underwent endoscopic submucosal dissection (ESD) for a large early-stage gastric cancer in the antrum, with the postoperative mucosal defect having covered approximately four-fifths of the circumference ( Fig. 1 ). Approximately 1 month later, she experienced gastric outlet obstruction, presenting with upper abdominal pain and postprandial vomiting. Gastroscopy revealed significant food retention, deformation of the gastric antrum, and scar formation ( Fig. 2 **a–c** ). It was barely possible to pass the endoscope through the deformed area ( Fig. 2 **d** ).

**Fig. 1:**
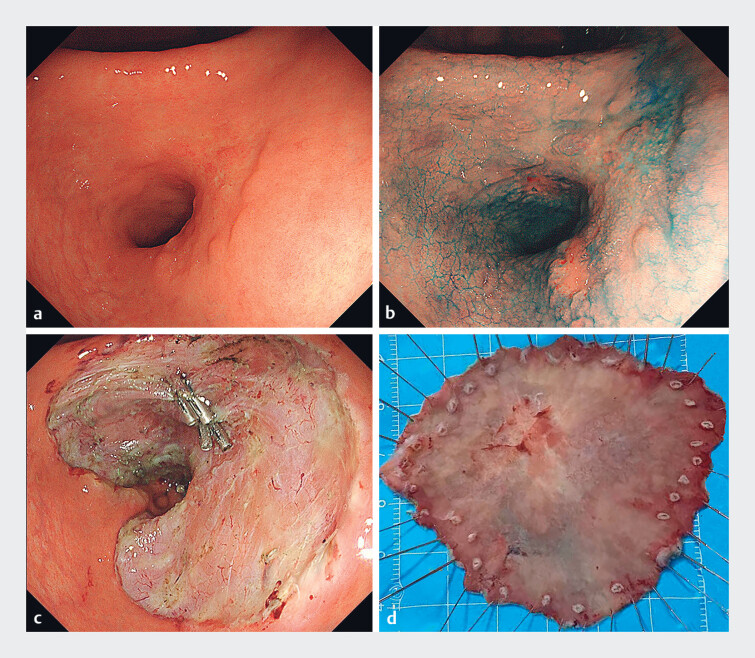
Images from the initial endoscopic submucosal dissection (ESD) procedure showing: **a** a large early-stage gastric cancer located in the antrum; **b** the lesionʼs morphology and boundaries clearly defined after indigo carmine staining; **c** a post-ESD mucosal defect involving over four-fifths of the circumference of the antrum; **d** the resected specimen, which measured approximately 70 × 55 mm.

**Fig. 2:**
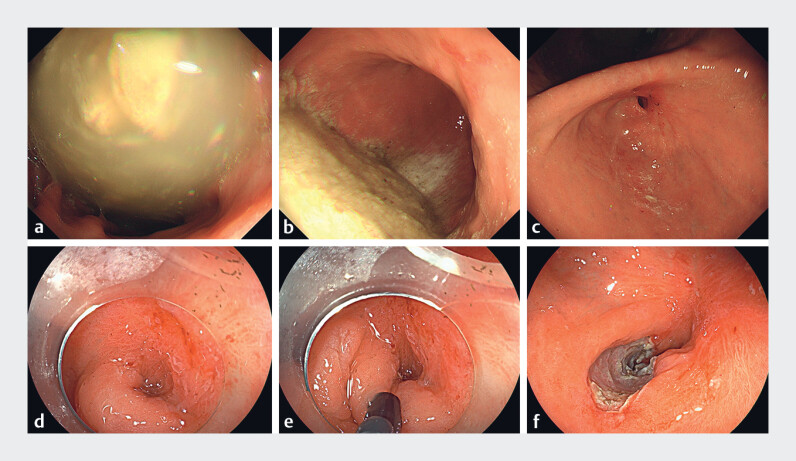
Gastroscopic images after the patient had developed gastric outlet obstruction showing: **a, b** substantial food retention in the stomach; **c, d** deformation of the gastric antrum with accompanying scar formation; **e, f** endoscopic septum division being performed on the anterior wall, with a longitudinal linear incision of 3–4 cm made through the mucosa and submucosa using an IT knife.

The patient was admitted and, after having been fasted, underwent gastrointestinal decompression. An endoscopic septum division was performed on the anterior wall, creating a longitudinal linear incision of 3–4 cm in length through the mucosa and submucosa using an IT knife (KD-611L; Olympus, Japan) ( Video 1 ). This procedure alleviated the obstruction, allowing smooth passage of the endoscope ( Fig. 2 **e, f** ), with the surgery taking only 10 minutes. The patient was discharged on the third postoperative day, with there being no recurrence of her obstructive symptoms during follow-up. Gastroscopy performed 2 months later showed significant improvement in the antral deformation ( Fig. 3 ).

Endoscopic septum division is performed to treat a nonstenotic gastric outlet obstruction following endoscopic submucosal dissection of a large cancer in the gastric antrum.Video 1

**Fig. 3:**
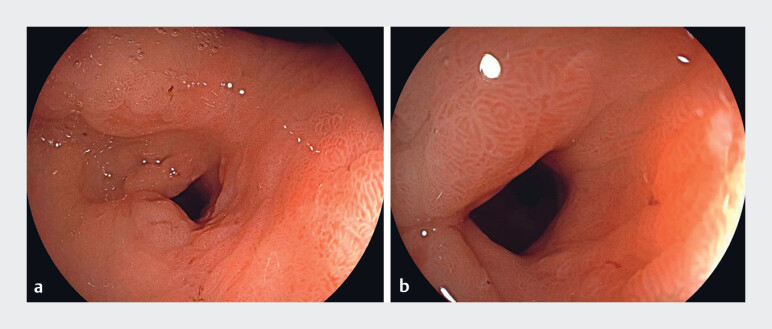
Gastroscopic images 2 months postoperatively showing significant improvement in the antral stenosis.

Mucosal defects that exceed three-quarters of the gastric antrum or pyloric canal are known risk factors for post-ESD stenosis 1 . Deformation of the gastric antrum is a well-known complication of extensive mucosal defects following ESD, and can result in gastric retention. Common treatments include endoscopic balloon dilation or local steroid injection, but these often require multiple procedures and carry risks of perforation or steroid-related complications 2 3 . Some studies have addressed the obstruction by performing reverse-traction ESD on the opposite side of the scar 4 . In our case, the post-ESD deformation likely resulted from scar formation, which retracted the opposing relaxed mucosa into a septum, thereby obstructing passage of food. We performed endoscopic septum division to release the obstruction by creating reverse traction. This technique is straightforward and time-efficient. To our knowledge, this is the first reported case of the use of endoscopic septum division to treat nonstenotic post-ESD gastric outlet obstruction, providing a reference for similar cases in future clinical practice.

Endoscopy_UCTN_Code_CPL_1AH_2AZ_3AD

## Notes

Endoscopy E-Videos https://eref.thieme.de/e-videos
*E-Videos* is an open access online section of the journal *Endoscopy* , reporting on interesting cases and new techniques in gastroenterological endoscopy. All papers include a high-quality video and are published with a Creative Commons CC-BY license. Endoscopy E-Videos qualify for HINARI discounts and waivers and eligibility is automatically checked during the submission process. We grant 100% waivers to articles whose corresponding authors are based in Group A countries and 50% waivers to those who are based in Group B countries as classified by Research4Life (see: https://www.research4life.org/access/eligibility/ ). This section has its own submission website at https://mc.manuscriptcentral.com/e-videos .
